# Top-hits for H1N1pdm Identified by Virtual Screening Using Ensemble-based Docking

**DOI:** 10.1371/currents.RRN1030

**Published:** 2011-01-03

**Authors:** Hung Nguyen, Ly Le, Thanh N. Truong

**Affiliations:** Department of Chemistry, University of Utah

## Abstract

A list of 27 promising antiviral drugs is proposed for use against the H1N1pdm strain. Since the binding site of the H1N1pdm neuraminidase is similar to that of the bird flu H5N1, an effective means to quickly identify top candidates for use against H1N1pdm is to use known bird-flu drugs and the 27 compounds from the NCI diversity set which bind best to H5N1 neuraminidase. These compounds serve as viable candidates for docking against the H1N1pdm neuraminidase, using ensembles extracted from molecular dynamics simulations of the H1N1pdm system. The ranking order of these top candidates was found to be different from the previously published results for H5N1. The results indicated that the Oseltamivir (Tamiflu) and Peramivir drugs have higher ranking than Zanamivir (Relenza). However, six drug candidates were found to bind more effectively to H1N1pdm neuraminidase than Tamiflu. Detailed hydrogen bond network analysis for these six candidates is also provided.

## **1. Introduction**

      The 2009 swine flu virus (H1N1pdm), which can be transmitted from human to human, has become a threat to the global health and economy.[1] [2] The World Health Organization declared the first flu pandemic in 41 years on June 11th, 2009. The current seasonal flu vaccine, which targets a different H1N1 strain, provides little or no protection against H1N1pdm.[3] In terms of medication, the two FDA approved antiviral drugs, Tamiflu (oseltamivir) and Relenza (zanamivir), are effective against this new type of virus.[1] [4] Unfortunately, such virus types are known for their quick mutations and gene assortments which enable them to escape the host immune systems and resist drugs. In specific, a case of swine flu resistant to Tamiflu was observed in Denmark on June 29, 2009 for the first time, and soon after that in Japan and Hong Kong.[5] Developing new vaccinations and antiviral drugs for this new H1N1pdm virus is an extremely urgent matter.

      Excluding the transmembrane and unstructured linker regions, the new swine and avian virus strains share 91.48% sequence identity of neuraminidase, a glycoprotein supporting release of new virions from infected cells to healthy ones.[6] The 3D structures of H1N1pdmand avian H5N1 neuraminidase have also revealed high similarity in their 150-loop and Sialic acid binding sites.[6] It is a reason why the Tamiflu developed for the H5N1 virus is also effective against H1N1pdm. These facts suggest top drug candidates for the H1N1pdm are also similar to those for the H5N1. The 27 top hits for H5N1 neuraminidase were suggested by Cheng et al.[7] from performing full virtual screening on H5N1 neuraminidase using the diversity set from the National Cancer Institute (NCI) small molecule database utilizing the ensemble-based docking approach.  

      In this study, we used the list of top hits from the study of Cheng et al.[7] and known bird flu drugs as the starting point for searching for promising antiviral drugs against the H1N1 pdm virus, and used the same ensemble-based docking approach as used by Cheng et al. This would be the shortest time to solution.  The selected ligands were docked against an ensemble of molecular models of H1N1pdm neuraminidase taken from a 20 ns molecular dynamics (MD) simulation.[6] The molecular model of H1N1pdm neuraminidase was built using H5N1 neuraminidase (PDB entry 2HU4) as the starting point then mutating corresponding residues. The detailed discussion on the H1N1pdm neuraminidase model can be found in our previous publication in PLoS .[6]

## **2. Materials and Methods**

### **2.1 *RMSD clustering to extract receptor ensembles from an all-atom MD simulation***

      The holo system with oseltamivir (Tamiflu) removed was used for RMSD clustering and the docking experiments.[6] Clustering analyses were performed on 20 ns MD trajectories using the g_cluster tool in the Gromacs package[8], as described by Daura et al.[9] In brief, snapshots at every 10 ps over the 20 ns simulation were recorded. 2000 resulting structures were superimposed, using all C_α_ atoms to remove possible rotational and translational movements of the whole system. We have visually verified that the binding-site residues of avian H5N1 neuraminidase, which cover the active site and are responsible for interaction with putative inhibitors, defined in Cheng et al.,[7] can also be used for the H1N1pdm neuraminidase. The RMSD clustering analysis was performed on this subset (117-119, 133-138, 146-152, 156, 179, 180, 196-200, 223-228, 243-247, 277, 278, 293, 295, 344-347, 368, 401, 402, and 426-441) using all-atoms (including side chains and hydrogens) with the cutoff of 1.5 Å. A total of 13 representative clusters were obtained, which account for 96.2% of the configuration space from the 20 ns MD trajectories.  These 13 clusters were used as receptors in the docking experiments.

**Figure:**
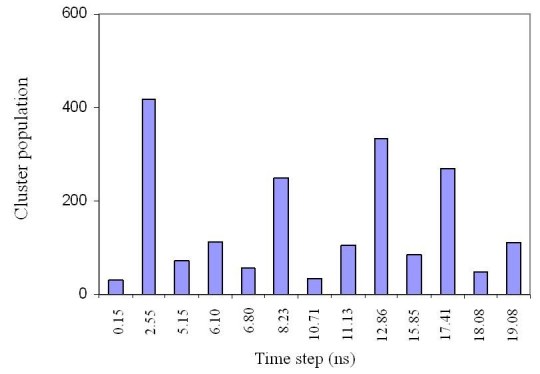
**Figure 1**. 13 representative ensembles accounting for 96.2% of the configuration space resulted from clustering analysis are ordered by the corresponding MD time step. The four largest cluster ensembles account for over 66% of the configuration space from the 20 ns MD simulation.

**Figure:**
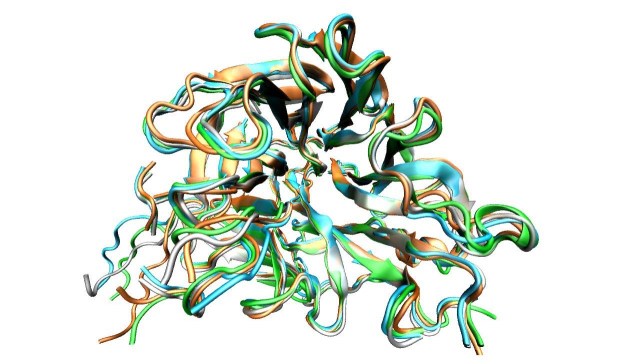
**Figure 2**. Overlapping structures of the four most populated clusters in Fig. 1. The most dominant cluster is colored in orange, the second one in silver, the third one in green and the fourth one in cyan.  

**
 
**


### **2.2* Molecular docking***

      In the docking experiments, the 13 most representative configurations were used as receptors. AutoDockTools 1.5.2[10] was used to add polar hydrogens, assign Gasteiger charges[11] and create grid binding boxes. The volume of each grid box was 72 x 72 x 72, with the default 0.375 Å spacing.  The binding box was positioned to encompass all three possible binding sites, namely the sialic acid, 150 and 430 cavities. AutoGrid version 4.2.1 was used to calculate the binding affinities using the following atom types: A (aromatic carbon), C, N, NA (hydrogen bond accepting N), OA (hydrogen bond accepting O), P, S, SA (hydrogen bond accepting S), Cl, HD (polar hydrogen) and e (electrostatics).

      The ligand set includes the 27 top hit compounds from Cheng et al.[7] and six additional compounds for reference purpose, namely: SA (sialic acid, N-acetyl neuraminic acid, aka, NANA or Neu5Ac), DANA (2,3-didehydro-2-deoxy-N-acetyl neuraminic acid), oseltamivir (Tamiflu)[12], zanamivir (Relenza)[13], peramivir (a clinical trial inhibitor)[14] and SK (shikimic acid, widely used as a chiral building block in the synthesis of Tamiflu)[15]. AutoDockTools version 1.5.2 was also used to merge nonpolar hydrogens, add Gasteiger charges and visually set up rotatable bonds for each ligand via AutoTors.

      Lamarckian genetic algorithm was used to do the docking experiments using AutoDock 4.2.1.[16] Docking parameters were chosen to reproduce structures of 13 corresponding oseltamivir–NA complexes in the MD simulation. The parameters are as follows: trials of 100 dockings, population size of 200, random starting position and conformation, translation step range of 2.0 Å, rotation step range of 50 degrees, maximum number of generations of 27000, elitism of 1, mutation rate of 2%, crossover rate of 80%, local search rate of 6%, 8 million energy evaluations, unbound model was “same as bound”, and docked conformations were clustered with the tolerance of 2.0 Å RMSD.

      Docking results were sorted by the lowest binding energy of the most populated cluster in cases of convergence. In the case of no dominant cluster, docking results were visually analyzed using VMD[17] to choose the best binding pose.

### **2.3 *Hydrogen bond analysis***

      Hydrogen bond analysis utilized a distance and angle cutoff of 3.5 Å and 45 degrees, respectively. The appearance frequency (AF) of important hydrogen bonds is calculated as follows:

\begin{equation*}AF=\frac{\sum_{i=1}^{13}{n_{i} h_{i}}}{\sum_{i=1}^{13}{n_{i}}}\end{equation*}

where i is the index number of each ensemble; n_i_ the size of each ensemble; and h_i_ = 1 if hydrogen bond exists, and 0 if otherwise.

### **2.4 *Statistical analysis of binding energy***

      After the dockings, statistical calculations were performed to obtain the final binding energies for compound ranking using the arithmetic mean (AM) and harmonic mean (HM) binding energies as defined in the previous study[7].   

      The arithmetic means were calculated directly from the binding energies

\begin{equation*}AM=\frac{\sum_{i=1}^{13}{n_{i} E_{i}}}{\sum_{i=1}^{13}{n_{i}}}\end{equation*}

where E_i_ is the binding energy of each ensemble with the standard deviation

_\begin{equation*}SD=\sqrt{\frac{\sum_{i=1}^{13}{n_{i}(E_{i}-AM)^{2} }}{\sum_{i=1}^{13}{n_{i}}}}\end{equation*}_

The harmonic means were calculated by first converting the binding energies into inhibition constant K_i_

_\begin{equation*}K_{i}= e^{\frac{1000E_{i} }{RT}}\end{equation*}_

where R is the Boltzmann constant and T is the temperature (298.15K)

The harmonic means \begin{equation*}\bar{K_{i}}\end{equation*}were calculated using:

\begin{equation*}\bar{K_{i}}=\frac{\sum_{i=1}^{13}{n_{i}}}{\sum_{i=1}^{13}{\frac{n_{i}}{K_{i}(i)}}}\end{equation*}

then the calculated \begin{equation*}\bar{K_{i}}\end{equation*} were converted back to HM binding energies.

## **3. Results and Discussion**

### **3.1 *Comparing binding modes of oseltamivir from molecular docking and MD simulation***

      First, we compare the binding modes of oseltamivir to the H1N1pdm neuraminidase resulted from the present docking experiment and those from previous full all-atom MD simulation. By applying the ensemble-based docking approach suggested by McCammon and co-workers,[7] [18] [19] [20] docking results are in good agreement with those from MD simulation for oseltamivir. As shown in Fig. 3, the two average structures are nearly superimposed except the pentyl group. Also shown are residues, which frequently interact with oseltamivir via hydrogen bonds: E119, D151, R292, and R371. One main reason that its binding free energy is less than that in the H5N1 case, as discussed below, is the loss of contact with R118.

**Figure:**
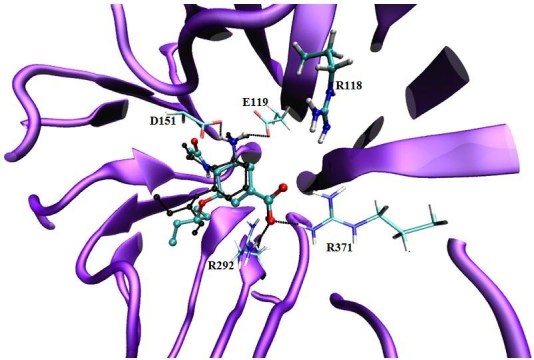
**Figure 3. **Oseltamivir docked in SA cavity of H1N1pdm neuraminidase as compared to that from MD simulation, as shown in black with no hydrogen.

      As shown in Table 1 below, the maximum all-atom RMSD between the two docking and MD ligand poses is less than 2 Å, and the average RMSD is of only 1.5 Å. The difference between the poses lies mainly in the orientation of the pentyl ether group, which interacts with the receptor primarily via hydrophobic forces. When excluding this group from the analysis, the average RMSD drops to below 1.1 Å. This comparison validates the accuracy of the ensemble-based docking approach.  Docking results over 13 receptor ensembles also show consistent statistics with the most populated cluster having the lowest binding energy and the largest percent population of the ligand cluster is found in the ensemble with largest percent population in the receptor space (see ensemble 1 as compared to ensemble 13).

**Table 1**. RMSD of the binding structures of oseltamivir (Tamiflu) from docking results in the 13 receptor ensembles compared to those extracted from the 20ns MD simulation. Also listed are the percent of population of the receptor ensemble, the percent contribution of the ensemble in the configuration space of the 20 ns MD simulation, and the percent population of the largest docked oseltamivir cluster.

**Figure:**
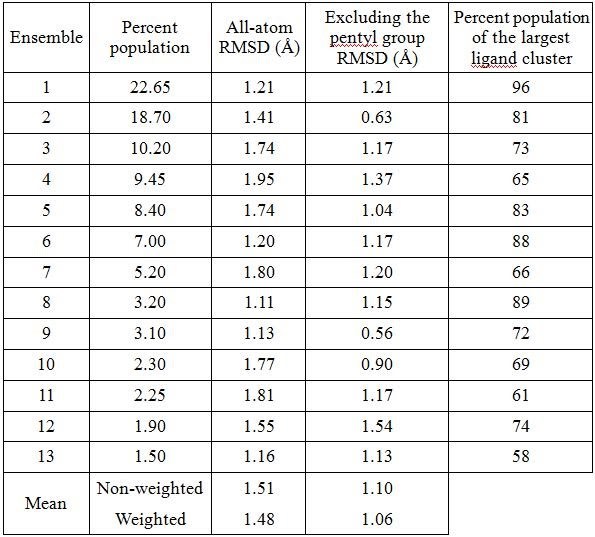

### **3.2 *Ranking of the top hit compounds***

      The ranking is made according to the harmonic mean of binding free energy instead of the arithmetic mean, since it gives more weight to the more populated clusters. The better performance of the harmonic mean of binding free energy is also shown in previous studies.[21] [22]

**Figure:**
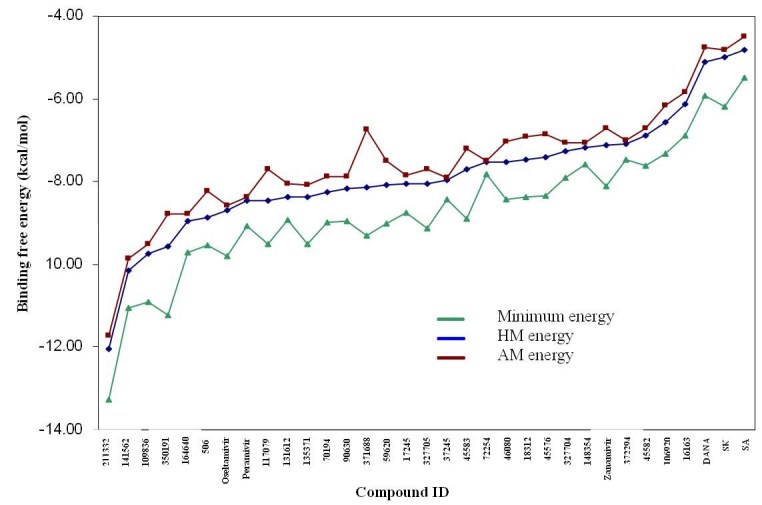
**Figure 4. **Binding energies of 33 compounds are listed with their NSC ID. Minimum energy is the lowest binding free energy docking into all 13 receptor ensembles. HM and AM energy are the harmonic mean and arithmetic mean binding free energy, respectively.

      First, the present ranking results for these top hits against H1N1pdm are quite different from those reported by Cheng et al. for H5N1 neuraminidase[7]. As shown in Table 2, some compounds rise (**2**, **5** and ** 6** as compared to the ranks of 16, 20 and 14, respectively for H5N1) while others move down (**29**, **19** and **25** as compared to 6, 3 and 9, respectively) in the ranking list. Oseltamivir retains its relatively high rank (with new/old rank of 7/4) while the changes in the rank for paramivir and zanamivir are substantial (peramivir jumps from 26 to **8**, right after oseltamivir, and zanamivir falls from 5 to **26**). Still, there are compounds such as **1**, **3**, ** 10** and **13** whose ranks are relatively unchanged. There are two possible reasons for the observed differences in the ranking order for the top hits calculated here for H1N1pdm as compared to those in the Cheng et al. study for H5N1. One is the differences in the hydrogen-bond networks in H1N1pdm and H5N1 systems, as discussed in more detail below, and another may be due to the improvement in the free energy scoring function in AutoDock 4.2.1. It is important to point out that the current approach may miss candidates in the NCI diversity set that should be in the top candidate list. Since the binding cavities of H1N1 pdm and H5N1 are very similar, the number of such candidates should be rather small.  However, to identify such candidates a complete screening of the diversity set should be done.

**Table 2**. Docking results for 33 compounds ranked by harmonic mean of binding energy. The first column is the final rank and also the compound ID. Also listed are the old rank in Cheng et al.[7], the predicted K_i_ calculated according to the harmonic mean binding free energies, as well as the arithmetic mean binding free energies and their corresponding standard deviation.

**Figure:**
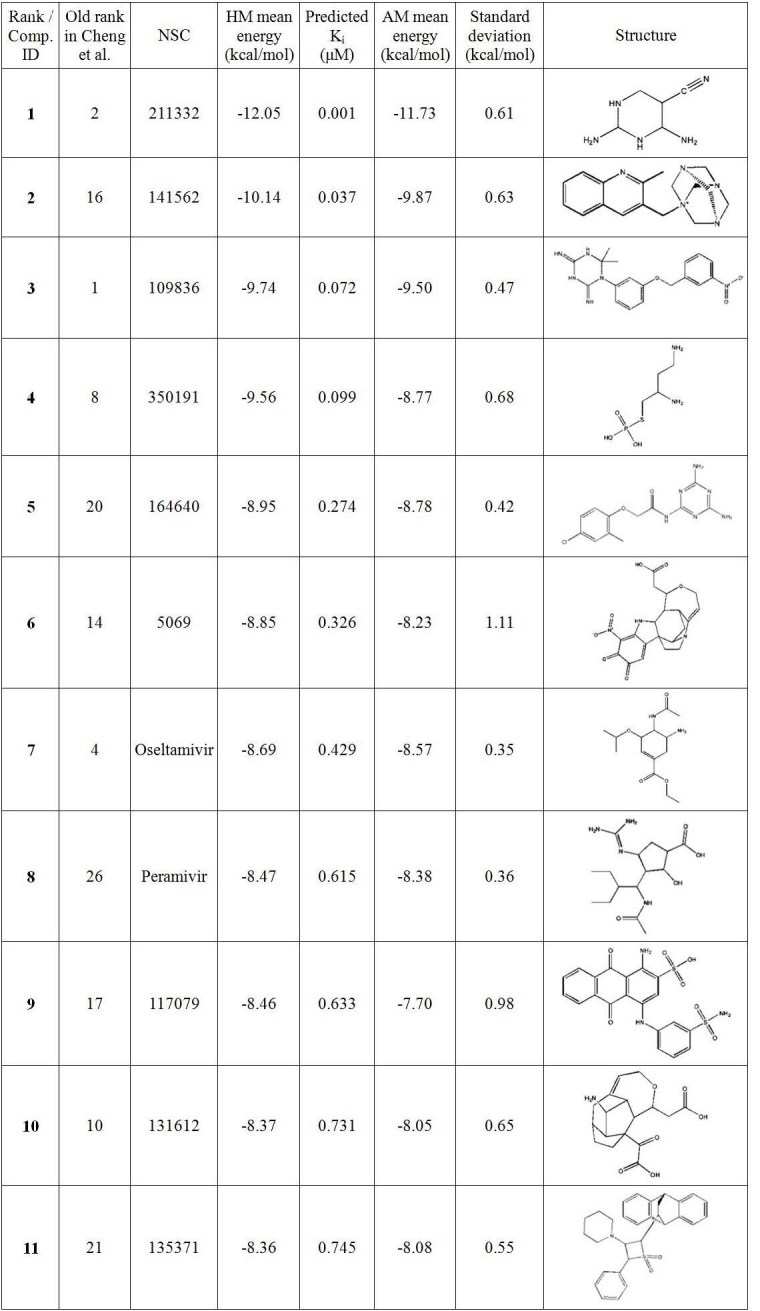

**Figure:**
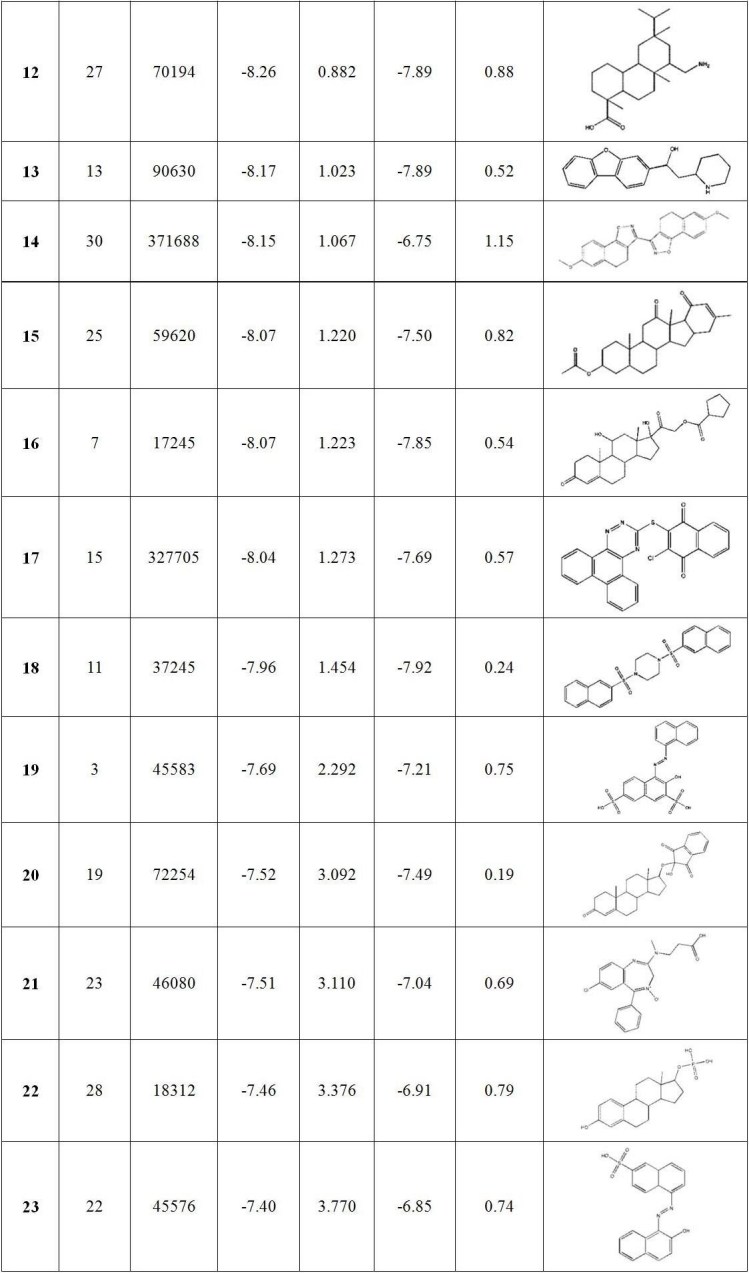

**Figure:**
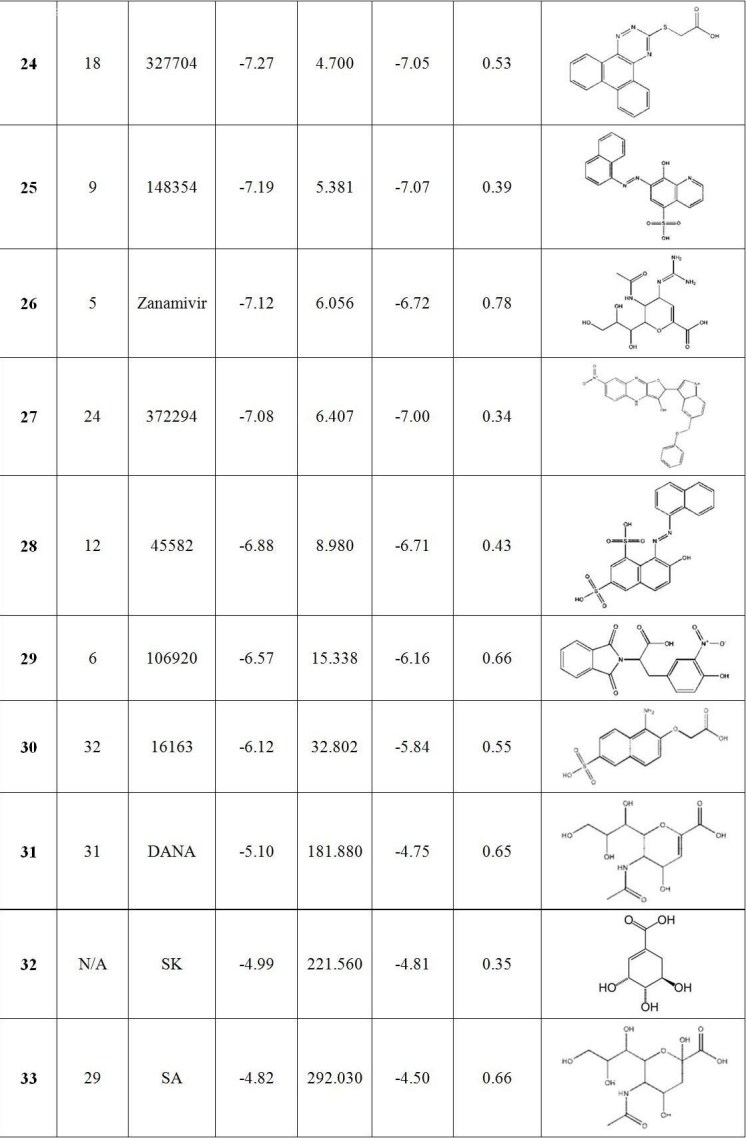

**Figure:**
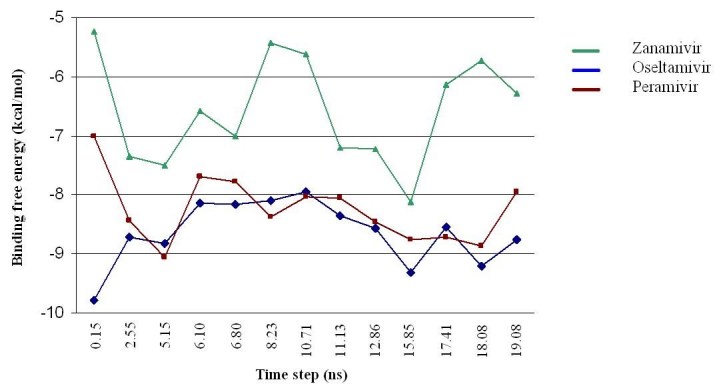
**Figure 5**. Binding energy spectra vs simulation time of zanamivir, oseltamivir and peramivir.

    Figure 5 shows the binding energy spectra of the three known bird flu drugs, zanamivir, oseltamivir and peramivir along the MD simulation time of H1N1pdm. Specifically it is the plot of the binding energies of these ligands with each of the H1N1pdm cluster ensembles. The results show zanamivir is consistently having smaller binding energy compared to the other two drugs.  A particular interesting result here is the prediction of zanamivir to be much less effective against H1N1pdm than peramivir whereas previous Cheng et al. study[7] predicted that it is more effective for treating H5N1.  However, more accurate simulations would be needed to confirm the relative binding affinity of these drugs. The present calculated result also confirms the observed effectiveness of oseltamivir (Tamiflu) for treating both H1N1pdm and H5N1. This confirmation not only provides another validation on the accuracy of the ensemble-based docking approach used in this study but also demonstrates the capability of the virtual screening technique in general.

### **3.3 *Hydrogen bond analysis of top binding compounds***

      Consistent with results from the recent MD simulation,[6] docking results also show that oseltamivir forms strong hydrogen bonds with E119, D151, R292 and R371.  In particular, the amino (NH_3_^+^) group of oseltamivir forms strong hydrogen-bonds with the carboxylate (COO^-^) groups of E119 and D151, whereas its carboxylate group forms hydrogen bonds with the guanidinium (NHC(=NH_2_^+^)NH_2_) groups of R292 and R371. These hydrogen bonds are highly conserved in all 13 receptor ensembles. The guanidinium group of R152 and the carboxylate groups of R227 and R277 sometimes form hydrogen bonds with the amide group of oseltamivir (Table 3, Figure 3).

**Table 3**. The frequency of hydrogen bond (in percent) with binding site residues for known drugs and top binding compounds utilizing the hydrogen bond distance and angle cutoffs of 3.5Ǻ and 45^0^, respectively.

**Table:** 

Residues	**1**	**2**	**3**	**4**	**5**	**6**	Oseltamivir	Peramivir	Zanamivir
E119	100	0	97	100	22	14	100	100	41
D151	97	0	14	100	0	0	98	98	47
R152	0	0	0	0	6	60	42	19	63
R156	0	100	94	0	71	8	0	0	0
W178	49	0	33	6	40	0	0	22	10
E227	98	0	0	94	16	0	13	100	100
E277	51	0	0	96	3	46	27	100	74
R292	0	0	97	100	3	72	100	100	100
R371	0	0	97	100	0	76	100	100	100
Y406	31	0	0	70	3	4	0	23	0

      Peramivir and zanamivir, like oseltamivir, also form highly conserved hydrogen bonds with the guanidinium groups of R292 and R371 through their carboxylate groups (Table 3, Figure 6). Peramivir’s guanidinium group also has strong contacts via hydrogen bonding and electrostatic interaction with carboxylates of E119, D151, E227 and E277; however, zanamivir does not. Its guanidinium still makes hydrogen bonds with these residues but less frequently as compared to oseltamivir and peramivir. This is perhaps one main reason for its smaller binding free energy (less negative) to the H1N1pdm neuraminidase. Another reason may be due to the receptors used in the docking procedure taken from the oseltamivir-bound holo structures, which possibly could not have good induced-fit effects towards zanamivir.

****

**Figure:**
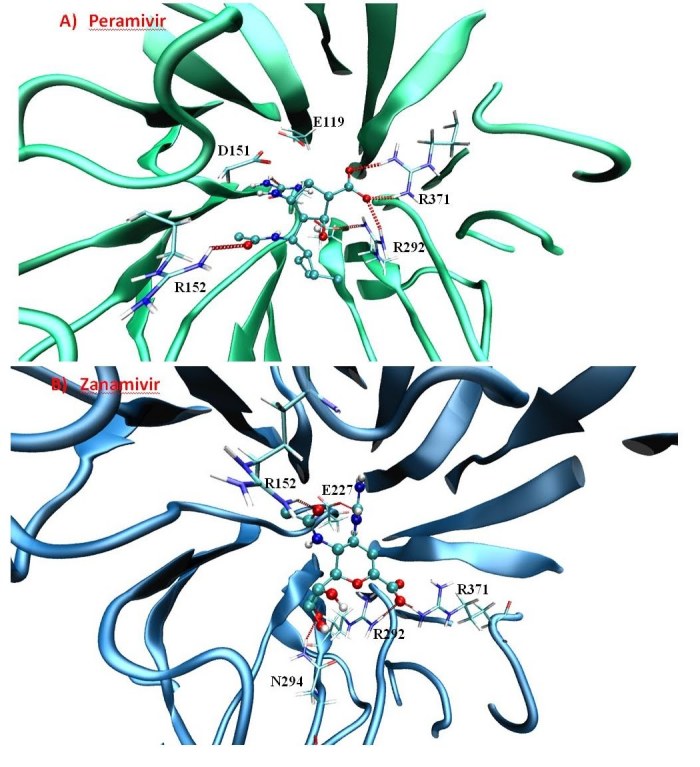
Figure 6. Peramivir (A) and zanamivir (B) docked in the A/H1N1 neuraminidase structure.  

      Compound ** 1**, which has a pyrimidinecarbonitrile framework, binds tightly in the SA cavity in all 13 receptor ensembles regardless of their flexibility. This compound not only forms hydrogen bonds with E119 and E227 reported in a recent study on avian H5N1 neuraminidase[7], but also forms strong ones with D151, and weak ones with W178, E277 and Y406 (Table 3, Figure 7A). This can explain its extremely high binding energy and thus its high ranking.

      Compound ** 3** (Figure 7C), on the other hand, binds less effectively to H1N1pdm than H5N1 neuraminidase due to the loss of interaction between its nitro (NO_2_) and guanidinium of R118.

      The guanidinium groups of the three residues (R118, R292 and R371) in the swine flu H1N1pdm neuraminidase are not in close proximity like those in H5N1. Therefore, several compounds including **2**, oseltamivir, peramivir and zanamivir lose part of their binding affinity since in H5N1 they usually form stronger hydrogen bonds with this trio.

      The special case is **2**, which has a tetraazatricyclo ring, and therefore it can form hydrogen bonds only with the guanidinium group of R156. Its binding energy is mainly due to electrostatic interactions with surrounding negatively charged carboxylate groups. 

**Figure:**
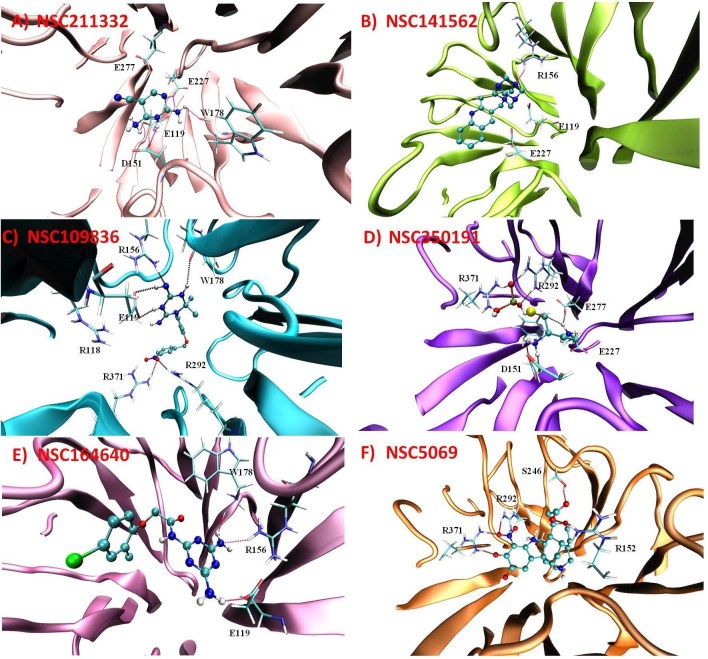
**Figure 7**. Docked structure of top-hits against swine flu neuraminidase (shown in CPK). A: NSC211332 (**1**); B: NSC141562 (**2**); C: NSC109836 (**3**); D: NSC350191 (**4**); E: NSC164640 (**5**);  F: NSC5069 (**6**).  Some residues predicted to be important are also shown in sticks. 

## **4. Conclusion**

      Since the A/H1N1 neuraminidase primary structure is 91% similar to that of H5N1 and the active sites of both proteins are similar, this study used the top hit compounds for H5N1 as the starting point of the virtual screening process to find promising antiviral drugs for H1N1pdm using the ensemble-based docking technique.  All 33 compounds, including the top binding compounds to avian H5N1 neuraminidase from the NCI diversity set and known drugs, were docked to ensembles of 13 H1N1pdm1 configurations that represent more than 96% of the configuration space covered by a 20 ns MD simulation.  The obtained results reveal that 6 compounds, specifically NSC211332, NSC141562, NSC109836, NSC350191, NSC1644640, and NSC5069, have higher binding affinity to the H1N1pdm neuraminidase protein than oseltamivir (Tamiflu) does. The results also confirm the observed effectiveness of Tamiflu against both the H1N1pdm and H5N1 flu. Furthermore, the results predicted zanamivir to be less effective against H1N1pdm than the other two known drugs, oseltamivir and peramivir.  However, due to the limitation of the method employed here, such finding would require more accurate calculations to confirm.  Detailed analysis on the hydrogen bond network also reveals the nature of interaction between top binding candidates with the swine A/H1N1 neuraminidase protein. These results suggest the possibility of using the present suggested top hit compounds for further computational and experimental studies to design new antiviral drugs against H1N1pdm virus and its variants. Furthermore, a more complete virtual screening using the full NCI diversity set or larger sets from ZINC database should be done to identify drug candidates that may have been missed in this study.

## **Acknowledgments**

      We thank the Ho Chi Minh City government for their support of the Institute for Computational Science and Technology (ICST) and the ICST staffs for their technical help.

## **Funding information**

      The authors acknowledge ICST for computing resources. Ly Le was partially supported by the Vietnam Education Foundation.

## **Competing interests**

      The authors have declared that no competing interests exist.
